# Treatment of Postpartum Myofascial Perineal Pain and Dyspareunia Through Local Anaesthetic Infiltrations Compared to Anaesthetic and Corticosteroids: A Randomised Double-Blind Clinical Trial †

**DOI:** 10.3390/jcm14093228

**Published:** 2025-05-06

**Authors:** Juan Antonio Solano Calvo, Jesús Manuel Barreiro García, Jerónimo González Hinojosa, Juan José Delgado Espeja, Antonio Rodríguez Miguel, Álvaro Zapico Goñi

**Affiliations:** 1 Obstetrics and Gynaecology Department, Hospital Universitario Príncipe de Asturias, Alcalá de Henares, 28805 Madrid, Spain; jeronimo.gonzalez@uah.es (J.G.H.); juanjose.delgado@uah.es (J.J.D.E.); alvaro.zapico@uah.es (Á.Z.G.); 2 Medical Science Department, University of Alcalá de Henares, Alcalá de Henares, 28801 Madrid, Spain; antonio.rodriguezmig@uah.es

**Keywords:** infiltration, myofascial pelvic pain, postpartum

## Abstract

**Background/Objectives**: The objective is to assess if transvaginal infiltration with anaesthetic only is non-inferior compared to anaesthetic plus corticosteroid for the treatment of myofascial pelvic pain. **Methods**: A randomised, double-blind, parallel-group (1:1) clinical trial was set at the Department of Obstetrics and Gynaecology, Hospital Universitario “Príncipe de Asturias” from December 2017 to June 2023. Women presenting myofascial perineal pain ≥ 4 on the visual analogue scale (VAS) 2 months after delivery, with instrumental delivery or prolonged second stage (>3 h) or foetal weight > 4000 g, were randomised into two groups to receive levobupivacaine 5 mg/mL or levobupivacaine 5 mg/mL plus betamethasone 3 mg/mL. For each trigger point detected, a transvaginal infiltration was performed using the corresponding treatment. The patients were followed up to 6 months. The primary endpoint was a change in the VAS score from baseline at 6 months. **Results**: A total of 114 women were enrolled, with 57 randomly assigned to each group. The median (IQR) VAS 2 weeks after infiltration decreased by a similar magnitude: median (IQR) 2 (1–3) in the levobupivacaine group and 2 (1–4) in the levobupivacaine + betamethasone group (*p*-value = 0.33). The same trend was observed at 6 months: median (IQR) 1 (1–4) in the levobupivacaine group and 1 (1–2) in the levobupivacaine + betamethasone group (*p*-value = 0.85). **Conclusions**: This study provides evidence that the use of anaesthetic-only infiltration is non-inferior compared to anaesthetic plus corticosteroid for the treatment of myofascial perineal pain.

## 1. Introduction

Pelvic and perineal pain is described as pain relating to the area of the pelvic bones or the reproductive organs. It is a frequent sequela after delivery and has been reported to affect up to 60% of women 3 months after childbirth [1,2]. Dyspareunia is also present in 25–50% of women during this period [1,2,3]. While most cases will resolve within the first 6 months, some will persist [4], affecting 6.1% of women 2 years postpartum [5].

Myofascial pelvic pain is characterised by the presence of myofascial trigger points in the pelvic floor structures, leading to hypertonic muscular areas and associated pain [6,7,8,9,10,11]. Although many theories have been proposed thus far, the most widely accepted hypothesis suggests dysfunction in the muscle motor endplate as the origin of myofascial pain [6,8,10,12,13,14]. This pain may also extend to other areas by propagation and convergence phenomena, including the presence of autonomic symptoms [15].

Some of the risk factors associated with postpartum perineal pain are multiparity, instrumental delivery, maternal age, prolonged second stage, episiotomy, and foetal weight over 4000 g. In the presence of these risk factors, some researchers advocate assessing and managing perineal pain early after delivery [16,17,18,19,20]. If untreated, a not insignificant number of patients could experience centralisation of pain [13]. Certain changes in the spinal cord and the brain cortex, including peripheral sensitisation (due to the proliferation of sodium channels), central sensitisation (due to N-methyl-D-aspartate activation and cortical reorganisation), diminished descending neuronal pain inhibition, and chronic immune system activation, are involved in the centralisation of pain and its perpetuation over time [12,13,15,21,22,23].

Pelvic pain is a major element in postpartum sexual dysfunction. It has been reported that up to 67% of patients present sexual dysfunction 3 months after delivery, with persistence in 14.9% of them. This is a devastating sequela for mothers that could lead to relationship problems or the lack of acceptance of the newborn, thus worsening their quality of life [23,24]. This should be evaluated with validated questionnaires [25,26].

Information on the treatment of myofascial pelvic pain is limited. Medical therapies available include oral analgesics, topical anaesthetics, and local infiltrations, although non-medical remedies may also be used, such as manual physiotherapy, acupuncture, and ice application [4,7,14,27,28,29,30].

Local infiltrations are an effective treatment with a good safety profile and acceptability. Two infiltration modalities have been described, transvaginal and transperineal, although the transvaginal method is more precise [31]. Needling can be dry or with the infiltration of anaesthetics alone or combined with corticosteroids or botulinum toxin, with similar results [14,27,32,33].

Both modalities seem to have good results. However, a combination with corticosteroids increases both treatment costs and the possibility of adverse effects. To our knowledge, no studies comparing the results of infiltrations using anaesthetics alone or combined with corticosteroids have been performed. In this study, we aimed to assess the role of corticosteroids in transvaginal injections for the treatment of myofascial pelvic pain in the postpartum period.

## 2. Methods

### 2.1. Design and Procedures

A randomised, double-blind, parallel-group (1:1) clinical trial was conducted to compare transvaginal infiltration with levobupivacaine alone against transvaginal infiltration with levobupivacaine plus betamethasone for the treatment of myofascial perineal pain at 6 months postpartum.

The patients were recruited at the Hospital Universitario “Príncipe de Asturias” Department of Obstetrics and Gynaecology (Madrid, Spain) from December 2017 to June 2023. The eligible women were those presenting with a myofascial perineal pain score of ≥4 on the visual analogue scale (VAS) [4], with instrumental delivery, prolonged second stage (>3 h), or foetal weight > 4000 g, with or without episiotomy. Women at <37 weeks of pregnancy at delivery, allergy to any of the drugs used in this study or metals, active vaginal infection, active malignant tumour, coagulopathy, trypanophobia, caesarean section delivery, pudendal neuralgia, or difficulty in understanding the informed consent were excluded.

All the women who fulfilled the conditions related to the delivery were referred to the Pelvic Floor Unit 2 months after delivery, where the eligible criteria were assessed. Myofascial perineal pain was identified through clinical interview and examination. Single-digit vaginal examination was performed as follows: the two fascicles of the levator ani muscle (iliococcygeus and pubococcygeus) and the coccygeus muscle were palpated clockwise, searching for trigger points. During the examination, myofascial perineal pain was assessed using a VAS from 0 to 10, where 10 corresponded to the worst pain imaginable and 0 to total absence of pain [4]. Women fulfilling the inclusion criteria were invited to enrol in the trial and given the informed consent form.

Once accepted, the patients were randomised into two treatment groups to receive levobupivacaine 5 mg/mL or levobupivacaine 5 mg/mL plus betamethasone 3 mg/mL. The randomisation sequence was performed and kept by the Pharmacology Unit of the hospital following a permuted block technique. The research team was not permitted to access it, ensuring that exposure was balanced across treatment arms and that the randomisation scheme was unpredictable and blind. The allocated treatments were placed in opaque envelopes identified with an alphanumeric code and delivered to the nursing team, which was agnostic to the study protocol. The medication was prepared in a covered syringe to ensure masking to the medical team and the patient.

The patients enrolled attended the Pelvic Floor Unit to perform four trial visits, according to the following schedule:

- Visit 1 (2 months after delivery): Clinical interview and physical examination. If the patient met the inclusion criteria, they were invited to enrol in this study. They were given an informed consent form and a validated scale of sexual function in Spanish (Female Sexual Function Index, FSFI) [26].
- Visit 2 (7 days after visit 1): Informed consent and the FSFI were collected. The patients were randomised to the double-blind treatment. The nursing team prepared the medication in a covered syringe, so neither the medical team nor the patient was able to see the medication administered. The infiltration was performed. The data were collected in the database.
- Visit 3 (15 days after visit 2): Physical examination and evaluation of pain with the VAS scale. If the pain persisted, a second infiltration or oral analgesia was offered. The data were collected in the database.
- Visit 4 (6 months after delivery): Physical examination and evaluation of pain with the VAS scale. The FSFI was re-administered. If the pain persisted, other therapies were offered following clinical guidelines. The data were collected in the database. End of follow-up.

All the variables of interest such as the sociodemographic data, clinical features, and treatments were prospectively collected and recorded in a specific electronic case report form.

### 2.2. Treatments

When a patient entered this study, the nursing team opened the corresponding randomisation envelope and prepared the medication using a completely opaque syringe.

A physical examination was carried out in the lithotomy position to identify potential trigger points. For each trigger point detected, a transvaginal injection was given using a 70 cm injeTAK adjustable needle (Medical Measurement, Palex Medical S.A., Barcelona, Spain) at a depth of 5 mm, infiltrating 2 cm^3^ of the corresponding treatment. After the procedure, haemostasis was checked.

Rescue treatment with dexketoprofen 25 mg was also offered to all the patients after the procedure. Use of this treatment was recorded in the database.

### 2.3. Efficacy and Safety

The primary endpoint of this study was a change in the VAS score from baseline 6 months postpartum.

Secondary endpoints included a change in the FSFI score from baseline to 6 months postpartum; safety, including the need for rescue analgesia after the first infiltration and at 6 months after delivery; and treatment failure.

Treatment safety was evaluated by recording all the serious adverse effects reported by the patients in the database.

### 2.4. Statistical Analysis

The sample size was calculated assuming a non-inferiority margin of 10%, an alpha error of 0.05, and a beta error of 0.2 and was estimated at 248 patients (124 for each group). An interim analysis was planned by the first quarter of 2023.

The quantitative variables were expressed as the mean and standard deviation (SD) or as the median and interquartile range (IQR) when the values were not normally distributed. The qualitative variables were expressed as number (n) and percentage (%). We used the Student *t*-test to compare two means or the Mann–Whitney U test as the non-parametric alternative. The chi-squared test was used to compare qualitative variables or Fisher’s exact test when the assumptions for the former were not fulfilled.

An intention-to-treat (ITT) analysis was performed to obtain the main estimators of the efficacy and safety, while the per-protocol effects were obtained as a secondary analysis. To manage loss to follow-up leading to missing values, we applied the last observation carried forward (LOCF) technique.

The statistical analyses were performed using STATA/MP (6 cores) v.17 (StataCorp, 4905 Lakeway Drive. College Station, TX 77845, USA). The statistical significance was set at *p* < 0.05.

### 2.5. Trial Registration

This clinical trial was registered in EudraCT with number 2017-004833-10 (https://www.clinicaltrialsregister.eu/ctr-search/search?query=2017-004833-10; accesed on 13 January 2025).

### 2.6. Writing

**Figure 1:**
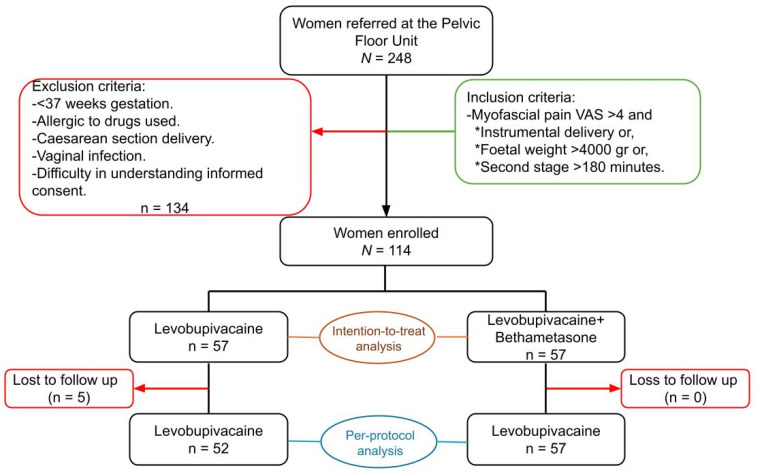
CONSORT flowchart showing the inclusion/exclusion criteria, recruitment, and randomisation into two groups. It also shows the patients lost to follow-up in each group and the final composition. VAS: visual analogue scale.

The CONSORT checklist was followed during the writing of this article and the CONSORT flowchart is presented (Figure 1).

## 3. Results

### 3.1. Baseline Characteristics of the Trial Population

A total of 248 women were referred to the Pelvic Floor Unit after delivery; 134 women did not fulfil the eligibility criteria so were excluded. Finally, a total of 114 women agreed to participate and enrolled in the trial, with 57 randomly assigned to levobupivacaine alone and 57 to levobupivacaine plus betamethasone (Figure 1).

The randomisation scheme created comparable groups within all the levels of the covariates at baseline, especially of those potentially associated with the outcomes of interest, such as history of gynaecological surgery, parity, dilation and labour time, delivery, episiotomy, vaginal tears, and foetal weight. In this regard, 46 (80.7%) women in the levobupivacaine group and 49 (86%) in the levobupivacaine + betamethasone group were primiparous, while 46 (80.7%) and 50 (87.7%), respectively, were instrumental deliveries, and 51 (89.5%) and 46 (80.7%) women, respectively, had undergone an episiotomy (Table 1).

After randomisation, five participants in the levobupivacaine group were lost to follow-up, as they no longer attended the trial visits and all attempts to establish telephone contact were unsuccessful (Figure 1).

### 3.2. Primary Efficacy Endpoint (Visual Analogue Scale)

The results from the interim ITT analysis showed equal effects on change in the VAS score at 6 months postpartum for both treatment arms and, consequently, the trial was prematurely interrupted.

**Figure 2:**
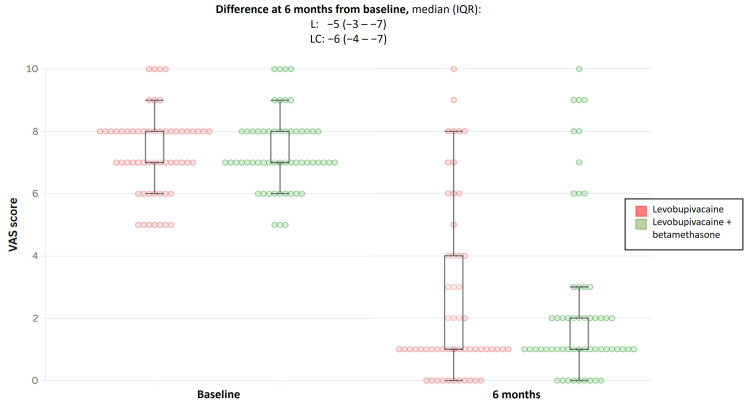
Boxplot showing difference in VAS score at 6 months from baseline for both treatment groups. VAS, visual analogue scale; L, levobupivacaine; LC, levobupivacaine + betamethasone; and IQR, interquartile range.

The median (IQR) VAS at entry was comparable between the two groups. At week 2 after the first infiltration, the VAS score decreased by a similar magnitude between the treatment arms (*p*-value = 0.33). At 6 months, the same trend was observed; the VAS score continued to decrease, albeit by a similar magnitude between the treatment arms, leading to a comparable difference between the groups from baseline: median (IQR) was 5 (3–7) for levobupivacaine and 6 (4–7) for levobupivacaine + betamethasone. The comparison was not statistically significant (*p*-value = 0.67; Table 2 and Figure 2). 

Little change was observed in these results following the per-protocol analysis.

### 3.3. Female Sexual Function Index (FSFI)

At baseline, 20 (35.1%) women in the levobupivacaine group and 28 (49.1%) women in the levobupivacaine + betamethasone group did not resume sexual intercourse after delivery, so the FSFI score was not obtained (*p*-value = 0.13; Table 3). Among the remaining women, the median total FSFI score differed slightly between the treatment arms, although the IQR overlapped completely: 16.5 (14.3–22.3) and 21.0 (14.7–25.2) for levobupivacaine alone and levobupivacaine + betamethasone, respectively (Table 3). This FSFI at baseline reflected the low scores observed across all the dimensions evaluated. The largest differences between the groups (although not statistically significant) were those for arousal and pain, with better scores in the levobupivacaine + betamethasone group for both parameters (Table 3). At 6 months after delivery, six (10.5%) women in the levobupivacaine group and nine (15.8%) in the levobupivacaine + betamethasone group had not yet had sexual intercourse. The total FSFI score improved notably within both treatment arms from baseline but by the same magnitude; the comparison was not statistically significant (Table 3). While the median difference from baseline for the total FSFI score seemed different, with a tendency to a greater score increase in the levobupivacaine group, the IQR for both overlapped (Table 3). 

FSFI improvement at 6 months was also observed for all the dimensions assessed, resulting in more balanced scores between the treatment arms, including arousal and pain. Notably, the difference in the score for the pain domain of the FSFI at 6 months from baseline yielded statistically significant although not clinically relevant results since the IQR for the difference included the null; the median (IQR) was 0.8 (0–2.9) for levobupivacaine and 0 (−0.8–2.4) for levobupivacaine + betamethasone (*p*-value = 0.03; Table 3).

### 3.4. Safety

At visit 3, 13 (22.8%) and 21 (36.8%) women met the criteria to receive rescue analgesia (*p*-value = 0.10). Of these, 7 (53.9%) women in the levobupivacaine group and 12 (57.1%) in the levobupivacaine + betamethasone group received a second infiltration of the same treatment (Table 4). However, six (46.1%) women in the levobupivacaine group and nine (42.9%) women in the levobupivacaine + betamethasone group declined a second infiltration and consequently were prescribed dexketoprofen (25 mg) (Table 4). At 6 months post-delivery, two (3.51%) women in both groups continued on dexketoprofen, while six (10.5%) women in the levobupivacaine group and five (8.77%) in the levobupivacaine + betamethasone group were considered treatment failures, as they did not experience any change in the primary endpoint or their condition in some cases worsened (Table 4). This difference, although insignificant, was mainly forced by the LOCF method, so was not observed in the per-protocol analysis. 

The cause of the treatment failure was also recorded in most of the patients. This highlighted central sensitisation and myofascial pain caused by other entities as the main reasons for failure of the infiltration (Table 5).

No major adverse effects were recorded during the development of the clinical trial. In addition, no minor adverse effects were reported by the patients nor the professionals.

## 4. Discussion

### 4.1. Main Findings

This study shows that the use of infiltration with anaesthetic only is non-inferior compared to anaesthetic plus corticosteroid infiltration for the treatment of myofascial perineal pain. The pain score reported by the patients improved overall by 90.3%, highlighting the good results of the treatment (intervention). Our study shows improvement in the FSFI score in both groups, underscoring the importance of treating pain in the early postpartum period, as its consequences could affect quality of life.

### 4.2. Interpretation

Our findings support the use of infiltrations as an alternative for treating myofascial pelvic pain. The results demonstrate the safety of this treatment, as no side effects were reported, in line with the published literature [14,27,32,33]. This should be also transmitted to patients, thereby increasing confidence in the treatment. Although the patients in our study were only treated with infiltrations, the evidence suggests that multimodal management of myofascial pain should be adopted to further optimise patient outcomes [7,21,34,35,36], adding manual physiotherapy and psychological support [28].

Sexual function after delivery is influenced by many factors [2,37,38], but perineal pain is the main element of sexual dysfunction in the postpartum period [39]. While the literature suggests avoiding perineal trauma and episiotomy, this is not always possible during delivery, hence the importance of treating pain in this patient subgroup.

Several types of trigger point injections have been described [7,14,36]. Dry needling is not recommended as the use of local anaesthesia reduces the discomfort of the injection [7]. Similar results have been reported for botulinum toxin use and anaesthetic only but with increased costs for the former [7,40,41]. Others such as magnesium-based infiltrations showed no difference compared to anaesthetic [42]. Cummings et al. reported that it is the needling and not the product administered that releases the trigger point and improves pain [43]. We did not consider the comparison with placebo as some studies demonstrate the effectiveness of dry needling. However, systematic revisions do not recommend the use of dry needling because of soreness of the injection site and the use of local anaesthesia is recommended [7].

Given that the first infiltration can fail for technical reasons, we performed two infiltrations within a 2-week lapse before considering treatment as a failure. Previous studies employed weekly infiltrations over a 4-week period [44]. However, we believe that the persistence of pain after the second infiltration should prompt reconsideration of the diagnosis of myofascial pelvic pain.

The anti-inflammatory effects of corticosteroids are reported to play a role in the treatment of some types of pain. Interestingly, our findings do not support this notion [45]. Returning to myofascial pain genesis theories, inflammatory elements do not play a major role after the acute damage event. The origin of myofascial pain is in the dysfunction of the motor endplate, not the inflammatory events [6,7,8,15]. Furthermore, some authors propose that trigger point infiltration develops an inflammatory response, which stops the cycle of myofascial pain [7,14,46]. Additionally, corticosteroids have secondary effects to bear in mind, including skin and muscle atrophy, peripheral nerve damage, decreased collagen synthesis, and reduced tissue vascularisation. This justifies the advisability of avoiding the use of corticosteroids whenever possible [45]. Moreover, the use of corticosteroids in the infiltration increases the cost of treatment. Thus, corticosteroid avoidance may not only be a safer approach for patients but might also be a more cost-effective strategy. Nevertheless, no cost-effectiveness studies have yet been performed to confirm this.

Sometimes, perineal pain can improve over time after delivery. But, in some studies, pain persistence above two months is a risk indicator of chronic pain [32]. Based on the accumulated evidence available and our own findings, we highly recommend screening for perineal pain and sexual dysfunction during the postpartum period to enable early intervention. The prompt treatment of pain is crucial to prevent pain sensitisation and centralisation [12,13,15,21,22,23]. We also recommend the treatment of myofascial pain in the postpartum period using transvaginal infiltrations with local anaesthetic only, as the addition of corticosteroids did not demonstrate any additional benefit.

This study also guarantees safety as no major adverse effects were recorded or reported. In addition, no minor adverse effects such as atrophy in the case of use of corticoids were reported. But some bias could affect this parameter, as patients may not report such events.

Although our results are promising, more studies should be conducted to continue assessing the reproducibility, safety, and efficacy of transvaginal infiltrations using local anaesthetic only. Studies are also required in women outside of the postpartum period to assess the efficacy of this treatment modality for myofascial pain.

### 4.3. Strengths and Limitations

This is, to our knowledge, the first randomised trial to compare anaesthetic-only with anaesthetic plus corticosteroid infiltrations in the treatment of myofascial perineal pain during the postpartum period. No other study has evaluated the effect of infiltrations on sexual function using validated scales, opening an avenue for future research in this area. One of the strengths of this study is that randomisation guarantees the homogenisation of both groups. Additionally, its double-blind nature minimises the risk of performance, data analysis, and patient outcome reporting bias. The treatment was applied by a small number of medical doctors following a strict technique. Consequently, variability in technique performance and success was reduced and reproducibility assured. Moreover, the large-size sample strengthens these results.

One of the main limitations of this study is that pain is a subjective perception. Some bias related to this subjectivity may influence the patient assessments and scores. The same limitation applies in the assessment of sexual dysfunction, where other variables can also have a considerable influence. The education level can have a certain influence over the subjective perception of pain and the patient’s actions (e.g., use of analgesics); we could not assess this variable as it was not recorded in a high number of patients.

Another of the main limitations is the sample size. The initial estimated sample size was 248 patients, but after the interim analysis, this study was interrupted and only 114 patients were enrolled. This could limit the validity of the results.

## 5. Conclusions

In summary, our study supports the early treatment of myofascial pain in the postpartum period through transvaginal infiltrations with local anaesthetic. However, pain should be treated within the context of multimodal management, including physiotherapy and psychological support to achieve cure rates. 

This study emphasises the importance of treating pain to prevent sexual dysfunction in the postpartum period. Nevertheless, further studies are needed to support our findings.

## Figures and Tables

**Table 1:** Baseline characteristics at randomisation for both groups.

	Levobupivacaine (n = 57)	Levobupivacaine + Betamethasone (n = 57)	*p*-Value
**Age, in years, mean (SD)**	32.6 (4.1)	33.1 (5.7)	0.58
**Ethnicity, n (%):** **Caucasian** **Others**	43 (75.4) 14 (24.6)	50 (87.7) 7 (12.3)	0.15
**Level of education, n (%):** **Primary** **Secondary** **University** **Not recorded**	6 (10.5) 19 (33.3) 14 (24.6) 18 (31.6)	4 (7.02) 13 (22.8) 23 (40.4) 17 (29.8)	0.30
**BMI, mean (SD)**	26.0 (4.82)	25.3 (4.19)	0.46
History of gynaecological surgery, n (%) *	8 (14.0)	7 (12.3)	0.15
**Parity, n (%):** **Primiparous** **Multiparous**	46 (80.7) 11 (19.3)	49 (86.0) 8 (14.0)	0.62
**Gestational age, in weeks, mean (SD)**	39.8 (1.20)	39.7 (1.16)	0.58
**Dilation time, in minutes, median (IQR)**	420 (210–600)	360 (270–560)	0.97
**Labour time ≥ 3 h, n (%)**	36 (63.2)	32 (56.1)	0.45
**Delivery, n (%):** **Eutocic** **Instrumental**	11 (19.3) 46 (80.7)	7 (12.3) 50 (87.7)	0.44
**Episiotomy, n (%)**	51 (89.5)	46 (80.7)	0.29
**Foetal weight, in grams, mean (SD)**	3345.0 (469.7)	3314.0 (445.0)	0.72
**Vaginal tear, n (%)**	29 (50.9)	31 (54.4)	0.71
**Use of analgesia upon delivery, n (%)**	6 (10.5)	9 (15.8)	0.41

Abbreviations: BMI, body mass index; IQR, interquartile range; SD, standard deviation; and VAS, visual analogue scale. * Includes cervical conisation, curettage, caesarean delivery, mammoplasty, and myomectomy.

**Table 2:** Primary efficacy endpoint (visual analogue scale) for both groups.

	**Levobupivacaine** (n = 57)	Levobupivacaine + Betamethasone (n = 57)	*p*-Value
**VAS at entry, median (IQR)**	7 (7–8)	7 (7–8)	0.87
**VAS at 2 weeks after treatment, median (IQR)**	2 (1–3)	2 (1–4)	0.33
**Difference at 2 weeks after treatment from baseline, median (IQR)**	-5 (-4–-6)	-5 (-3–-6)	0.37
**VAS at 6 months, median (IQR)**	1 (1–4)	1 (1–2)	0.85
**Difference at 6 months from baseline, median (IQR)**	-5 (-3–-7)	-6 (-4–-7)	0.67

Abbreviations: IQR, interquartile range; VAS, visual analogue scale.

**Table 3:** Female Sexual Function Index (FSFI) scores from both treatment groups during the clinical trial.

	Levobupivacaine (n = 57)	Levobupivacaine + Betamethasone (n = 57)	*p*-Value
**FSFI at baseline, median (IQR):**			
**Total**	16.5 (14.3–22.3)	21.0 (14.7–25.2)	0.15
**Desire**	2.4 (1.8–3.6)	3.0 (2.4–3.6)	0.14
**Arousal**	2.7 (2.1–3.9)	3.6 (2.4–4.8)	0.09
**Lubrication**	3.3 (1.8–3.9)	3.6 (2.4–3.9)	0.71
**Orgasm**	3.6 (2.0–4.8)	3.2 (1.6–4.4)	0.76
**Satisfaction**	4.0 (2.4–4.8)	4.4 (3.2–4.8)	0.25
**Pain**	2.0 (1.2–2.7)	2.8 (1.6–4.4)	0.08
**No sexual intercourse, n (%)**	20 (35.1)	28 (49.1)	0.13
**FSFI at 6 months, median (IQR):**			
**Total**	26.7 (20.5–30.2)	25.7 (21.3–29.6)	0.70
**Desire**	3.6 (2.4–4.8)	3.6 (3.0–4.5)	0.71
**Arousal**	4.5 (3.3–5.1)	4.5 (3.6–5.4)	0.94
**Lubrication**	4.5 (3.6–5.4)	4.7 (3.6–5.4)	0.47
**Orgasm**	4.8 (3.6–5.6)	4.4 (3.2–5.6)	0.39
**Satisfaction**	4.8 (3.6–6.0)	4.8 (3.5–5.6)	0.86
**Pain**	4.8 (2.4–6.0)	3.8 (2.6–5.2)	0.29
**No sexual intercourse, n (%)**	6 (10.5)	9 (15.8)	0.41
**Difference in total score at 6 months, median (IQR)**	6.4 (0–12.1)	3.2 (0–10.3)	0.30
**Difference in pain score at 6 months, median (IQR)**	0.8 (0–2.9)	0 (-0.8–2.4)	0.03

Abbreviations: FSFI, Female Sexual Function Index; IQR, interquartile range.

**Table 4:** Use of rescue analgesia and treatment failure rate for both groups.

	Levobupivacaine (n = 57)	Levobupivacaine + Betamethasone (n = 57)	*p*-Value
**Rescue analgesia after first infiltration, n (%):**	13 (22.80)	21 (36.80)	0.26
**Received a second infiltration**	7 (53.90)	12 (57.10)	
**Received dexketoprofen**	6 (46.10)	9 (42.90)	
**Treatment failure, n (%):**			0.06
**No changes in VAS at 6 months from baseline**	4 (7.02)	0 (0)	
**Increased VAS at 6 months from baseline**	2 (3.51)	5 (8.77)	
**Rescue analgesia at 6 months, n (%):**	2 (3.51)	2 (3.51)	1.00

Abbreviations: VAS, visual analogue scale.

**Table 5:** Causes of infiltration technique failure in both treatment groups.

Group/Patient		Cause of Failure
**Levobupivacaine**	1	Central sensitisation.
	2	Not recorded.
	3	Levator ani muscle hypertonia.
	4	Coxalgia.
	5	Central sensitisation.
	6	New trigger point infiltrated outside the trial.
**Levobupivacaine** **+** **Bethametasone**	1	Vulvar neurinoma.
	2	Not recorded.
	3	Not recorded.
	4	Central sensitisation.
	5	Central sensitisation.

## Data Availability

The raw data supporting the conclusions of this article will be made available by the authors on request.

## Abbreviations

The following abbreviations are used in this manuscript:

VAS	Visual Analogue Scale
FSFI	Female Sexual Function Index
IQR	Interquartile Range
L	Levobupivacaine
LC	Levobupivacaine + Betamethasone
SD	Standard Deviation
